# Healing Potential of *Picrorhiza kurroa *(Scrofulariaceae) rhizomes against indomethacin-induced gastric ulceration: a mechanistic exploration.

**DOI:** 10.1186/1472-6882-8-3

**Published:** 2008-01-31

**Authors:** Debashish Banerjee, Biswanath Maity, Subrata K Nag, Sandip K Bandyopadhyay, Subrata Chattopadhyay

**Affiliations:** 1 Department of Biochemistry, Dr. B.C. Roy Post Graduate Institute of Basic Medical Sciences & IPGME&R, 244B, Acharya Jagadish Chandra Bose Road, Kolkata - 700 020, India; 2 Bio-Organic Division, Bhabha Atomic Research Centre, Mumbai - 400 085, India

## Abstract

**Background:**

The present study was undertaken to evaluate the potential of the rhizomes of the Indian medicinal plant, *Picrorhiza kurroa *in healing indomethacin-induced acute stomach ulceration in mice and examine its capacity to modulate oxidative stress and the levels of prostaglandin (PGE_2_) and EGF during the process.

**Methods:**

Male swiss albino mice, ulcerated with indomethacin (18 mg/kg, p. o., single dose) were treated up to 7 days with different doses of the methanol extract of *P. kurroa *rhizomes (designated as PK). The healing capacity of the most effective dose of PK (20 mg/kg, p. o. × 3 d) was compared with that of omeprazole (Omez) (3 mg/kg, p. o. × 3 d). The effects of the drug-treatment for one and three days on the biochemical parameters were assessed by comparing the results with that of untreated mice of the 1^st ^and 3^rd ^day of ulceration. The stomach tissues of the mice were used for the biochemical analysis.

**Results:**

The macroscopic indices revealed maximum ulceration on the 3^rd ^day after indomethacin administration, which was effectively healed by PK. Under the optimized treatment regime, PK and Omez reduced the ulcer indices by 45.1% (*P *< 0.01), and 76.3% respectively (*P *< 0.001), compared to the untreated ulcerated mice.

Compared to the ulcerated untreated mice, those treated with PK for 3 days showed decreased the levels of thiobarbituric acid reactive substances (TBARS) (32.7%, *P *< 0.05) and protein carbonyl (37.7%, *P *< 0.001), and increased mucin (42.2%, *P *< 0.01), mucosal PGE_2 _(21.4%, *P *< 0.05), and expressions of COX-1 and 2 (26.9% and 18.5%, *P *< 0.05), EGF (149.0%, *P *< 0.001) and VEGF (56.9%, *P *< 0.01). Omez reduced the TBARS (29.4%, *P *< 0.05), and protein carbonyl (38.9%, *P *< 0.001), and increased mucin (38.3%, *P *< 0.01), without altering the other parameters significantly.

**Conclusion:**

PK (20 mg/kg, p. o. × 3 days) could effectively heal indomethacin-induced stomach ulceration in mice by reducing oxidative stress, and promoting mucin secretion, prostaglandin synthesis and augmenting expressions of cyclooxygenase enzymes and growth factors.

## Background

Gastrointestinal toxicity associated with the nonsteroidal anti-inflammatory drugs (NSAIDs) is an important medical problem despite recent pharmaceutical advances [1]. Besides inducing gastric ulceration, the NSAIDs also appear to inhibit ulcer healing [2,3]. Consequently, prevention of gastrointestinal disorder continues to be of concern for both clinical practitioner and researchers. In spite of their efficacy in managing the NSAID induced gastric ulceration, the currently available synthetic anti-ulcer drugs confer side effects, and are also expensive especially for the rural population. To this end, we have reported promising anti-ulcerogenic activity of several plant products [4-6].

*Picrorhiza kurroa *(family: Scrofulariaceae, common name: Picrorhiza, katuka, kutki) is a small perennial herb growing in the hilly parts of the North-Western Himalayan region in India and Nepal. The leaf, bark and the underground parts of the plant, mainly rhizomes are widely used in the traditional Indian (Ayurvedic) systems of medicine since ancient times. Although it shows anti-oxidant, anti-inflammatory, and immunomodulatory activities, it is most valued for its hepatoprotective effect. The bitter rhizomes of *Picrorhiza *have been used for thousands of years in India to treat people with indigestion [7], and constipation due to insufficient digestive secretion [8]. *Picrorhiza *is considered as a trophorestorative herb for the liver, as well as a potent immune stimulant [9]. Its constituent, picroliv is also reported to possess choleretic effect [10], and prevent hepatic injury caused by ethanol [11], chemicals [12] and microorganism [13].

The primary aim of this investigation was to study the healing property of the methanol extract of *P. kurroa *rhizome (designated as PK) against indomethacin-induced acute gastric ulceration of mice *vis-à-vis *that of the commercial drug, omeprazole (Omez). Indomethacin, a representative of the nonsteroidal anti-inflammatory drug (NSAID) family, causes gastric ulcers through various processes, including generation of ROS, inhibition of prostaglandin synthesis, infiltration of polymorphonuclear leukocytes, induction of apoptosis, and initiation of lipid peroxidation [14,15]. Hence, the ulcer healing capacities of the drugs, assessed by histopathology, was correlated with their effects in reducing the indomethacin-induced oxidative stress. The antioxidant activity of the test samples was assayed in terms of their ability to protect oxidative damages to lipids, proteins, and thiol-dependent antioxidant defense in gastric tissues. Further, the NSAIDs-induced gastropathy is also attributed to the decreased synthesis of the prostaglandins [16]. Consequently, the role of the test samples in increasing mucus secretion, and expression of epidermal growth factor (EGF), as well as elevating PGE_2 _synthesis were also investigated.

## Methods

### Plant material

The rhizome of *P. kurroa *(designated as PK), procured from the local market was used for the present work. The plant was taxonomically identified (accession no. 324139) by the Botanical Survey of India, Indian Botanical Garden, Kokata, India.

### Chemicals

2-Thiobarbituric acid (TBA), ethanol, butanol and ethyl acetate were procured from E. Merck (Mumbai, India), while trichloroacetic acid (TCA) was from Thomas Baker (Mumbai, India). Alcian blue, indomethacin, bovine serum albumin (BSA), haematoxylene, alum, eosin, butylated hydroxytoluene (BHT), guanidine hydrochloride, trifluoroacetic acid (TFA), omeprazole (Omez), 3,3 '-diaminobenzidine (DAB), rabbit anti-mouse EGF and Trizma base were procured from Sigma Chemicals (St. Louis, MO, USA). Other reagents used were 35% hydrogen peroxide (Lancaster, Morecambe, U.K.), 2,4-dinitrophenyl hydrazine (DNPH), disodium hydrogen phosphate and sodium dihydrogen phosphate (BDH, Pool Dorset, U.K.), sucrose and 5,5'-dithiobis-2-nitrobenzioc acid (DTNB) (SRL, Mumbai, India), horse and goat serum (Banaglore Genie, Banaglore, India), PGE_2 _metabolite kit (Cayman Chemical, Michigan, USA) and peroxidase conjugated goat anti-rabbit IgG (EMD Biosciences, Sandiego, USA).

### Instrumentation

The absorbance spectrophotometry was carried out at 25°C using a Jasco V-550 UV-Vis spectrophotometer. Wavelength scans and absorbance measurements were made in 1 ml quartz cells of 1 cm path length.

### Preparation of plant extracts

The air-dried rhizomes of *P. kurroa *(250 g) were chopped into fine pieces, soaked in methanol (1 l) for two days and the supernatant decanted. After repeating the entire process three times, the combined extracts were filtered through a nylon mesh, and evaporated in vacuo and finally lyophilized. The crude extract, designated as PK throughout the manuscript, was stored in a vacuum dessicator.

### Preparation of the drugs

The drugs were prepared from PK and Omez as aqueous suspensions in 2% gum acacia as the vehicle, and administered to the mice orally.

### Pharmacological tests

#### Animals and experimental protocol for ulceration

Male swiss albino mice were bred at Dr. B. C. Roy Post Graduate Institute of Basic Medical Sciences, Kolkata, India and BARC Laboratory Animal House Facility, Mumbai, India. These were procured after obtaining clearance (Post Graduate Institute of Basic Medical Sciences, Kolkata Animal Ethics Committee 507/CPCSEA, Sanction No. IAEC/SB-2/2004/UCM-16, dated 06.15.04 and BARC Animal Ethics Committee (BAEC), laboratory animal facility. sanction no. BAEC/03/05, dated 11.07.05) from the respective Animal Ethics Committees of the two centres and were handled following International Animal Ethics Committee Guidelines. The 6–8 weeks old mice (25–30 g) were reared on a balanced laboratory diet as per NIN, Hyderabad, India and given tap water ad libitum. They were kept at 20 ± 2°C, 65–70% humidity, and day/night cycle (12 h/12 h). At the beginning of each experiment, all animals were identified by typical notches in the ear and limbs and then randomized. This was done to perform all the experiments in a blinded fashion. Ulceration in the mice was induced by administering indomethacin (18 mg/kg, p. o.) dissolved in distilled water and suspended in the vehicle, gum acacia (2%) as a single dose. Our studies with 5, 10, 15, 18, 20, 25 and 30 mg/kg, p. o. of indomethacin revealed that the lowest doses (5 and 10 mg/kg) provided minor ulceration after 6 h of its administration, while the higher doses (25 and 30 mg/kg) led to mortality. The chosen dose (18 mg/kg) produced optimal ulceration, with inflammation and mucosal insult, without causing any mortality to mice. The animals were deprived of food but had free access to tap water 24 h before ulcer induction.

#### Standardization of drug dose

For the standardization of drug doses, PK (5, 10, 20, and 40 mg/kg) was given as a single dose per day up to seven days. The first dose of PK and Omez were given 6 h after the indomethacin administration. In the subsequent six days, the test samples were given at 9 AM on each day. Five mice were taken in each group and each experiment was repeated three times. The mice were sacrificed on the 1^st^, 3^rd^, 5^th ^and 7^th ^days, 4 h after administering the last dose of PK. The extent of ulcer healing was assessed from the macroscopic damage scores (MDS) of the untreated and treated ulcerated mice on the respective days. The optimized treatment regime (drugs dose and period of treatment) were assessed from the MDS of the groups of mice receiving the above treatment.

#### Assessment of ulceration and healing from MDS

The mice were sacrificed after an overdose with thiopental. The stomach from the normal and treated groups were removed rapidly, opened along the greater curvature, and thoroughly rinsed with normal saline. The ulcerated gastric mucosal areas were visualized using a transparent sheet and a dissecting microscope. The MDS was assessed [17] by grading the gastric injury on a 0–4 scale, based on the severity of hyperemia and hemorrhagic lesions: 0 – almost normal mucosa, 0.5 – hyperemia, 1 – one or two lesions, 2 – severe lesions, 3 – very severe lesions, 4 – mucosa full of lesions. (lesions – hemorrhagic erosions, hyperemia – vascular congestions). The experiments were performed by two investigators blinded to the group and treatment of animals. The sections were coded to eliminate an observer bias. Data for the analyses are presented as the mean ± SEM from the review of a minimum of three sections per animal and five animals per group.

#### Studies on the biochemical parameters

The MDS results revealed maximum ulcer healing after three days of drug treatment. The stomach ulceration in the untreated mice also reached peak on the 3^rd ^day after indomethacin administration. Hence, we assessed the biochemical parameters under the optimized treatment regime [PK (20 mg/kg) and Omez (3 mg/kg)] up to the 3^rd ^day of ulceration only. For this, the following seven groups each containing 5 mice were selected from those used for the MDS assay and the data shown are derived from three replicates:

Group I – normal control mice; Group II – ulcerated mice, and sacrificed after 10 h (considered as one day); Group III – ulcerated mice, and sacrificed on the 3^rd ^day; Group IV-V – ulcerated mice, treated with PK and Omez respectively for 1 day, and sacrificed 4 h after administration of test samples; Group VI-VII – ulcerated mice, treated with PK and Omez respectively for 3 days, and sacrificed in the 3^rd ^day, 4 h after the last dose of the test samples.

#### Quantification of protein and lipid damages during ulceration and healing

The glandular stomach tissues were pooled from five animals for each tissue, rinsed with appropriate buffer and used for biochemical studies. The wet weights of the tissues were recorded and the experiments were carried out in triplicates. The glandular portions from the control, ulcerated and drug-treated mice taken at different time intervals were homogenized with a glass-teflon homogenizing tube in a 50 mM phosphate buffer, pH 7.4 and centrifuged at 1200 × g to obtain the supernatant. The amount of protein carbonyls in the tissue homogenate, was determined following a reported method [18]. DNPH (4 ml, 10 mM) in 2 M HCl was added to the supernatant (1.0 ml), which was incubated for 1 h with intermittent shaking. Ice-cold 20% aqueous TCA solution (5 ml) was added and the mixture incubated for 15 min. The precipitated protein was washed 3 times with ethanol-ethyl acetate (1:1), dissolved in 1 ml of a solution containing 6 M guanidine HCl in 20 mM potassium phosphate (monobasic) adjusted to pH 2.3 with trifluoroacetic acid. After centrifuging, the absorbance of the supernatant was read at 362 nm (∈ = 2.2 × 10^4 ^M^-1 ^cm^-1^).

For the analysis of lipid peroxidation (in terms of TBARS), a 10% homogenate from each of the respective samples was prepared in a buffer (320 mM sucrose, 5 mM HEPES, 20 mM EDTA and 0.01% BHT). These were subjected to differential centrifugation (1200 × g and 12000 × g) to obtain the mitochondrial pellets, which were washed with the buffer (150 mM KCl and 20 mM phosphate buffer) and finally suspended in a 50 mM phosphate buffer, pH 7.4. The mitochondrial membrane fraction (1 ml) was treated with TCA-TBA-HCl (2 ml, 15% TCA, 0.375% TBA, 0.25 N HCl) containing 0.01% BHT, heated on a boiling water bath for 15 min, cooled and centrifuged. The absorbance of the supernatant was measured at 535 nm (∈ = 1.56 × 10^5 ^M^-1 ^cm^-1^) [19].

#### Measurement of non-protein thiol (NP-TSH)

Following a reported method [20], the gastric mucosal NP-TSH was measured. Briefly, fundic stomach homogenates from the control, ulcerated, drug-treated mice were made in 0.2 M Tris-HCl buffer, pH 8.2 containing 20 mM EDTA and centrifuged. An aliquot of the homogenate (1 ml) was precipitated with ice-cold 20% TCA (1 ml) and centrifuged. The supernatant (1 ml) was added to 2 ml of 0.8 M Tris-HCl buffer, pH 9 containing 20 mM EDTA and mixed with 0.1 ml of 10 mM DTNB. The absorbance of the yellow chromogen at 412 nm (∈ = 13.6 × 10^4 ^M^-1 ^cm^-1^) was read.

#### Mucin assay

Following a reported method [21], the free mucin in the gastric tissues was estimated. Briefly, the glandular portion of the stomach was separated from the lumen of the stomach, weighed and transferred immediately to 10 ml of 0.1% w/v Alcian blue (Ab) solution (in 0.16 M sucrose solution buffered with 0.05 mM sodium acetate solution, pH adjusted to 5.8). After staining for 2 h, the excess dye was removed from the tissue by two successive rinses with 10 ml of 0.25 M sucrose solution. The dye complexed with the gastric wall mucus was extracted with 10 ml of 0.5 M magnesium chloride by intermittent shaking (1 min) at 30 min intervals for 2 h. The blue extract (2 ml) was vigorously shaken with an equal volume of diethyl ether. The resulting emulsion was centrifuged at 3600 rpm for 10 min, and the absorbance of the aqueous layer was read at 580 nm. The quantity of Ab extracted per g of wet glandular tissue was calculated from a standard curve prepared using various concentrations of Ab.

### COX-1 and COX-2 expression assay

Within 30 min of harvesting, the ulcerated portions of the stomach tissues were fixed in neutral-buffered 10% formol saline and sectioned. After 24 h of fixation followed by embedding in a paraffin block, it was cut into sections of 5 micron onto a glass slide and the expressions of COX-1 and COX-2 were quantified following a reported procedure [22], with slight modification. After deparaffinization in xylene, the sections were treated with a graded series of alcohol and subsequently rehydrated in PBS at pH 7.5. The sections were sequentially treated with 3% hydrogen peroxide in PBS, and protein blockers (5% bovine serum albumin, 5% normal goat serum in PBS), and incubated overnight at 4°C with primary antibody (mouse anti-COX-1 and COX 2, 1:200). After incubation for 2 h at room temperature with peroxidase conjugated rabbit anti-mouse secondary antibody (1:500), a positive reaction was detected by exposure to stable DAB for 2 to 5 min. In control sections, antibodies were omitted, and only PBS was added. After counterstaining the slides with Meyer's hematoxylin, the intensities of colored immunolocalized products were evaluated using Biovis MV500 software. Five areas from each section were scanned and the integrated optical density (IOD) in each area was calculated. The IOD of the negative control was subtracted from the IOD of each experimental section for each animal in all the groups.

#### Prostaglandin assay

Following harvesting of the stomach, the corpus (full thickness) was excised, weighed (100 mg) and suspended in 10 mM sodium phosphate buffer, pH 7.4 (1 ml). The tissue was minced finely with scissors and incubated at 37°C for 20 min. After centrifugation (9000×g), the prostaglandin E_2 _(PGE_2_) levels in the supernatant were measured by ELISA [23], and the concentrations are expressed as pg/mg protein.

### EGF expression assay

The immunostaining of EGF was carried out [22] following a similar method used for COX-1 and COX-2, with minor modifications. In this case, after blocking the endogenous peroxidase and treatment with the protein blockers, the tissue sections were incubated overnight at 4°C with primary antibody at the appropriate dilution. In control sections, only PBS was added omitting the antibodies. After incubation for 1 h at room temperature with peroxidase conjugated goat anti-rabbit IgG, a positive reaction was detected by exposure to DAB for 2 to 5 min. After counterstaining the slides with Meyer's hematoxylin, the intensities of colored immunolocalized products were quantified using Biovis MV500 software. Five areas from each section were scanned and the integrated optical density (IOD) in each area was calculated. The IOD of the negative control was subtracted from the IOD of each experimental section for each animal in all the groups.

#### Assay of serum VEGF

The serum VEGF was measured using the blood samples drawn from the descending aorta, with commercially available ELISA kits following manufacturer's instruction.

### Gastric secretory assay (pylorous ligation model)

To study the effects of the test drugs on gastric secretion, the pyloric ligation of the stomach standardized in our laboratory was performed. One group of mice (experimental control) received 2% gum acacia in distilled water (0.2 ml per mice, p.o.), while the different treatment groups were given PK (20 mg/kg × 3 d), Omez (3 mg/kg × 3 d), indomethacin (18 mg/kg × 1 d) as such or along with PK (20 mg/kg × 3 d), Omez (3 mg/kg × 3 d) respectively. On the 3^rd ^day, the abdomen of the animals was opened under light ether anesthesia for pyloric ligation (PL). After closure of the abdomen, the animals were put into cages under light restraint and allowed to recover from the anesthesia. After 6 h, the animals were sacrificed, the abdomen was opened and a ligature was placed around the esophagus. Stomach was removed and the contents were drained into a graduated centrifuge tube after making a small nick along the greater curvature adjacent to pyloric ligation. The gastric contents were collected, measured, centrifuged, and subjected to biochemical analysis.

### Statistical analysis

The data are presented as mean ± SEM. Parametric data which includes all the biochemical parameters were analyzed using a paired 't' test for the paired data or one way analysis of variance (ANOVA) followed by a Dunnet multiple comparisons post test. Nonparametric data (macroscopic scoring) were analyzed using Kruskal-Wallis test (nonparametric ANOVA) followed by a Dunn's multiple comparisons post test. A probability value of *P *< 0.05 was considered significant. The IC_50 _value of PK was estimated using the Probit analysis and the significance level of the analyses was investigated by the chi-square test.

## Results

### Extraction and phytochemical analysis

The extraction process yielded the methanol extract, PK in 12.57% w/w yield. The TLC analysis of PK revealed it to be a complex mixture of a large number of organic compounds, most of these being present as glycosides as revealed by Molisch's test. Presence of apocynin, andorsin, picrosides I and II, and katukoside along with other unidentified compounds in PK was confirmed by comparison of its TLC profile with those of the authentic samples.

### Standardization of dose of PK for gastric ulcer healing in mice

**Figure 1:**
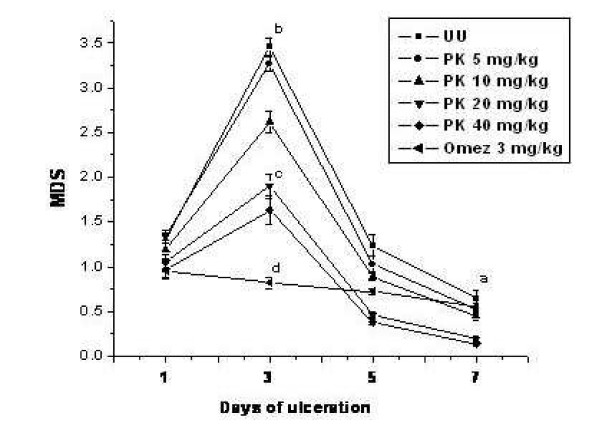
The time course of stomach ulceration and its prevention by different doses of PK. Stomach ulceration in mice was induced by oral administration of indomethacin (18 mg/kg body weight). Different doses of PK were used for the experiments. The ulcer indices were measured on different days 4 h after the last dose of PK and the values are mean ± S.E.M. (n = 15). ^a^*P *< 0.01, ^b^*P *< 0.001 compared to 1^st ^day ulcerated untreated mice; ^c^*P *< 0.01, ^d^*P *< 0.001 compared to the group III mice.

The dose of PK for effective ulcer healing were optimized by carrying out the treatment with PK (5, 10, 20 and 40 mg/kg) up to seven days and comparing the MDS values of the treated and untreated mice on the respective days. The time course of the extent of stomach ulceration (as revealed from MDS values) and its prevention by different doses of PK are shown in Fig. 1. The mice receiving vehicle only showed no mucosal lesions. Treatment of mice with indomethacin (18 mg/kg) produced typical time-dependent acute lesions in the gastric mucosa. The ulceration reached maximum on the 3^rd ^day of ulceration when the MDS value increased by 162.1% compared to that on day one (*P *< 0.001). Thereafter, there was a gradual recovery due to natural healing, and on the 7^th ^day after ulceration, the MDS value was reduced by 49.2% compared to that on day one (*P *< 0.01).

PK, at all the chosen doses showed maximum ulcer healing on the 3^rd ^day of ulceration, and the effect was dose-dependent. Compared to respective ulcerated controls, the MDS reductions (45.1%–52.9%, *P *< 0.01) by PK (20 and 40 mg/kg) were similar, and significantly better than that at its lower dose (10 mg/kg). The effect of PK (5 mg/kg) was insignificant. Under similar conditions, treatment with Omez (3 mg/kg) for 3 days reduced the MDS value by 76.3% (*P *< 0.001) compared to that of the group III mice. The healing observed on extending the treatment up to seven days with PK was better than that observed with the three-day treatment regime. However, a major part of this was due to autohealing, with less contribution by PK.

Overall, treatment with PK (20 mg/kg) and Omez (3 mg/kg) for 3 days after ulcer induction provided optimal ulcer healing. In view of these, all subsequent experiments were carried out with the same treatment regime. The chosen dose of Omez, which is also the recommended therapeutic dose for humans was optimized by us [6].

For the untreated mice, peak ulceration (maximum MDS) was observed on the third day of indomethacin administration. Hence, this time point was selected to find out the IC_50 _value of PK. Considering the MDS values of the 3^rd ^day ulcerated untreated mice as 100%, the IC_50 _value of PK was found to be 23.30 ± 3.50 mg/kg.

#### Qualitative assessment

The macroscopic examinations revealed that indomethacin administration led to a marked damage to the glandular portion of the mice gastric mucosa. Within 6 h following indomethacin administration, superficial erosion and mild inflammation in the stomach was observed indicating acute ulceration. On the day of ulcer induction, loss of foveolar structure along with cryptic architecture was the prominent feature in most of the untreated mice. Also, mild inflammatory infiltrate containing neutrophils was observed in the lamina propria. Treatment with both PK and Omez even for one day restricted the damage with patchy areas of denuded structural epithelium.

Indomethacin induced gastropathy became much pronounced on the third day showing punched out multiple areas of ulceration with inflammatory infiltrate containing neutrophils and macrophages in the mucosa, sub-mucosa and muscle coat along with hemorrhagic serosa. A large number of abnormal cells with altered nucleus to cytoplasm ratio were noticed. Treatment with PK and Omez for 3 days led to reduced number of inflammatory cells along with an increased number of healthy normal cells in the gastric mucosa, as well as submucosa, serosa and muscle layers with minimum mucosal congestion.

### Effect of PK and Omez on lipid peroxidation and protein oxidation in stomach tissue

The effects of indomethacin intake alone and following administration of PK and Omez on the extent of lipid peroxidation (measured in terms of TBARS), protein oxidation (measured in terms of protein carbonyl content), the thiol-dependent defense system (NP-TSH) and mucin content in the gastric tissues of mice are shown in Table 1 and Table 2.

**Table 1:** The effect of PK and Omez on the levels of TBARS, protein carbonyls, non-protein thiol, and mucin in the ulcerated gastric tissue of mice^a ^on day one of ulceration

Parameters	Group I mice normal control	Group II 1^st ^day ulcerated control	Group III PK-treated	Group IV Omez-treated
TBARS (nmoles/mg protein)	0.85 ± 0.03	1.18 ± 0.05^c^	0.94 ± 0.06^d^	0.89 ± 0.07^d^
protein carbonyls (nmoles/mg protein)	1.08 ± 0.05	1.62 ± 0.09^b^	1.49 ± 0.12	1.53 ± 0.07
NP-TSH (nmoles/mg tissue)	1.99 ± 0.09	1.66 ± 0.08^b^	1.86 ± 0.08	1.81 ± 0.09
mucin (μg/g tissue)	335.03 ± 14.43	213.02 ± 0.59^b^	240.33 ± 20.49	253.33 ± 14.53^d^

^a^Stomach ulceration in mice was induced by oral administration of indomethacin (18 mg/kg). PK (20 mg/kg/day × 1 day) and Omez (3 mg/kg/day × 1 day) were used as the test samples. The assays were carried out 4 h after the administration of the test samples. The values are mean ± SEM (n = 15). ^b^*P *< 0.05, ^c^*P *< 0.01 compared to normal mice; ^d^*P *< 0.05 compared to the group II mice.

**Table 2:** The effect of PK and Omez on the levels of TBARS, protein carbonyls, non-protein thiol, and mucin in the ulcerated gastric tissue of mice^a ^on day three of ulceration

Parameters	Group I normal control	Group III 3^rd ^day ulcerated control	Group V PK-treated	Group VI Omez-treated
TBARS (nmoles/mg protein)	1.02 ± 0.06	1.53 ± 0.06^c^	1.03 ± 0.09^d^	1.08 ± 0.05^d^
Protein carbonyls (nmoles/mg protein)	1.18 ± 0.07	2.61 ± 0.17^c^	1.62 ± 0.10^f^	1.59 ± 0.09^f^
NP-TSH (nmoles/mg tissue)	2.06 ± 0.09	1.73 ± 0.09	2.06 ± 0.14^d^	2.05 ± 0.1^d^
mucin (μg/g tissue)	343.30 ± 16.91	213.29 ± 17.91^b^	303.34 ± 17.4^e^	295.06 ± 15.62^e^

^a^Stomach ulceration in mice was induced by oral administration of indomethacin (18 mg/kg). PK (20 mg/kg/day × 3 days) and Omez (3 mg/kg/day × 3 days) were used as the test samples. The assays were carried out on day three of ulceration, 4 h after the last dose of the drug. The values are mean ± SEM (n = 15). ^b^*P *< 0.05, ^c^*P *0.001 compared to normal mice, ^d^*P *< 0.05, ^e^*P *< 0.01, ^f^*P *< 0.001 compared to the group III mice.

Indomethacin administration markedly stimulated lipid peroxidation in gastric tissues, elevating the TBARS content by 38.8% (*P *< 0.01) and 53% (*P *< 0.001) respectively on the 1^st ^and 3^rd ^day of ulceration, compared to that of the normal control mice. Treatment with PK and Omez showed immediate effect, reducing the TBARS content by 20.3% and 24.6% respectively compared to the group II mice (*P *< 0.05). Treatment with PK and Omez for three days reduced it by 32.7% and 29.4% compared to the group III mice (*P *< 0.05).

Compared to the normal value, the protein carbonyl content of the ulcerated mice was higher (50%) on the first day (*P *< 0.05) which increased by 120.3% at peak ulceration (*P *< 0.001). None of the test drugs showed immediate impact on day one. However, after treatment for three days, PK and Omez reduced the protein carbonyl contents by 37.7% and 38.9% compared to that of the group III mice (*P *< 0.001).

### Effect of PK and Omez on NP-TSH in stomach tissue

Indomethacin administration to mice depleted the NP-TSH level significantly on the day of ulcer induction itself compared to that in normal mice (16.6%, *P *< 0.05). However, there was no further reduction in the level at peak ulceration. Treatment with the PK and Omez for three days increased the NP-TSH content by 19.1% and 18.5% compared to that of the group III mice (*P *< 0.05).

### Effect of PK and Omez on mucin contents in stomach tissue

The indomethacin administration immediately reduced the mucin secretion in the ulcerated mice compared to that in normal mice (36.4%, *P *< 0.05) and the status remained unaltered even at peak ulceration. Compared to that of the group II mice, Omez showed immediate effect augmenting the mucin level by 18.9% (*P *< 0.05) while the effect of PK was less. Prolonging the treatment for three days augmented the mucin level significantly by 42.2% and 38.3% in both PK and Omez treated groups (*P *< 0.01).

### Autohealing of gastric ulceration and effect on biochemical parameters

We also followed natural recovery in absence of any treatment up to 7 days. The autohealing began five days after the ulcer induction but was more pronounced on the 7^th ^day, when noticeable regeneration atypia in the gastric gland was observed (Figure nor shown). This also suggested that the ulceration was acute. The rate of healing in the untreated mice was significantly slower compared to the drug-treated animals. The autohealing for 7 days was accompanied with reduction of TBARS (32%) and protein carbonyl (35%) along with increase in the mucin content (11%) compared to those in the group III mice (three day-untreated). The values were significantly less than that observed with the mice treated with PK and Omez for three days (*P *< 0.05).

### Effect of PK and Omez on COX isoforms

**Figure 2:**
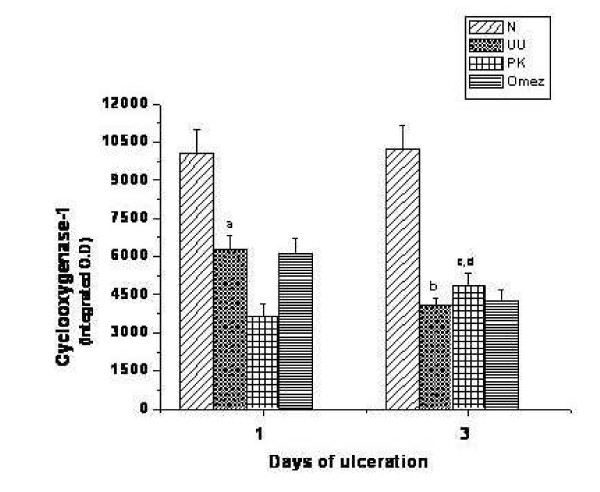
Comparative time dependent activity of PK and Omez in regulating the expression of tissue COX-1 isoform in acute gastric ulcerated mice. The COX-1 expression was quantified using Biovis MV500 software. Data are expressed as means ± SEM for fifteen mice. ^a^*P *< 0.05, ^b^*P *< 0.01 compared to normal mice, ^c^*P *< 0.05 compared to the respective ulcerated mice, ^d^*P *< 0.05 compared to the Omez-treated mice. N – normal mice, UU – ulcerated untreated mice, PK – ulcerated PK-treated mice, Omez – ulcerated Omez-treated mice.

**Figure 3:**
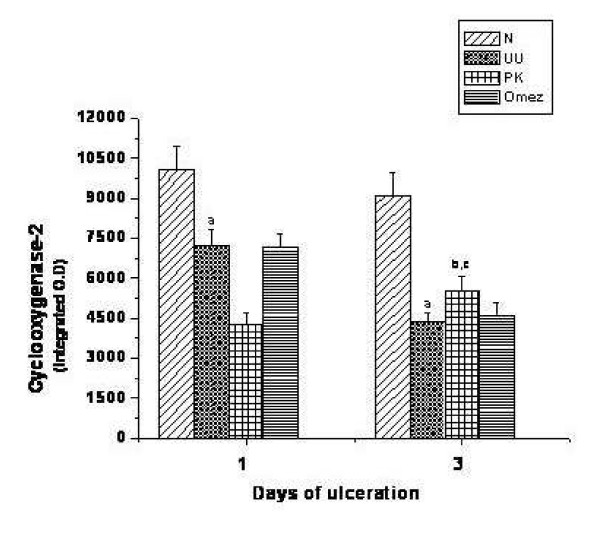
Comparative time dependent activity of PK and Omez in regulating the expression of tissue COX-2 isoform in acute gastric ulcerated mice. The COX-2 expression was quantified using Biovis MV500 software. Data are expressed as means ± SEM for fifteen mice. ^a^*P *< 0.01 compared to normal mice, ^b^*P *< 0.05 compared to the respective ulcerated mice, ^c^*P *< 0.05 compared to the Omez-treated mice. N – normal mice, UU – ulcerated untreated mice, PK – ulcerated PK-treated mice, Omez – ulcerated Omez-treated mice.

Quantification of the immunopositive areas for the COX isoforms revealed reduced expression of COX-1 (28.5%, *P *< 0.05), and COX-2 (37.6%, *P *< 0.01) in the ulcerated untreated mice on the day of ulcer induction itself, compared to normal mice (Fig. 2 and Fig. 3). At peak ulceration, the expressions of COX-1 and COX-2 were lowered by 52.1% and 54.3% respectively (*P *< 0.01) compared to the normal values. None of the test samples had any immediate effect on the expression of the mucosal COX isoforms. However, on prolonging the treatment for three days, PK increased the expression of COX-1 and COX-2 isoforms by 26.9% and 18.5% respectively compared to that in the group III mice (*P *< 0.05). The effect of PK was better (*P *< 0.05) than that of Omez, which increased expression of the enzymes insignificantly.

### Effect of PK and Omez on PGE_2 _level

**Figure 4:**
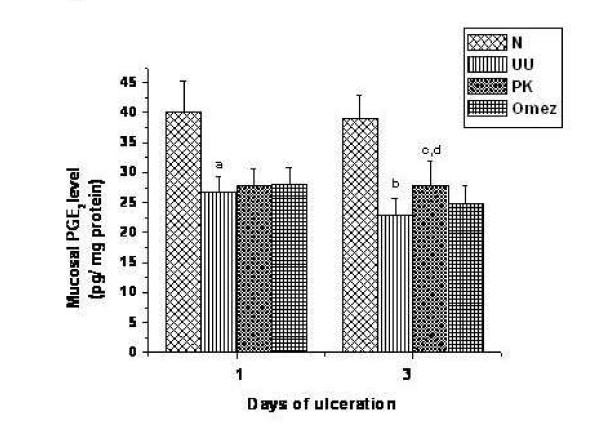
Comparative time dependent ability of PK and Omez in regulating PGE_2 _synthesis in acute gastric ulcerated mice. The PGE_2 _levels were measured using ELISA. Data are expressed as means ± S.E.M. for fifteen mice. ^a^*P *< 0.05, ^b^*P *< 0.01 compared to normal control mice; ^c^*P *< 0.05 compared to the respective ulcerated mice; ^d^*P *< 0.05 compared to the Omez-treated mice. N – normal mice, UU – ulcerated untreated mice, PK – ulcerated PK-treated mice, Omez – ulcerated Omez-treated mice.

The synthesis of mucosal PGE_2 _was markedly suppressed by 33.4% (*P *< 0.05) and 41.0% (*P *< 0.01) on the 1^st ^and 3^rd ^day of ulceration, compared to that in the normal mice. None of the test samples showed any immediate effect in modulating the PGE_2 _level. However, on prolonging the treatment for 3 days, the mucosal synthesis of PGE_2 _in the PK-treated mice increased significantly by 21.4% (*P *< 0.05) compared to that of the group III mice. The effect of PK was significantly (*P *< 0.05) better than that of Omez, which increased the PGE_2 _level marginally (8.3%). The results are summarized in Fig. 4.

### Effect of PK and Omez on EGF expression

**Figure 5:**
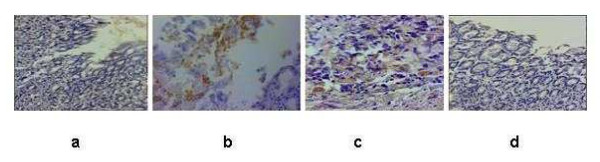
The changes in the tissue EGF expression due to acute gastric ulceration of mice and its regulation by PK and Omez on the 3^rd ^day of ulceration. The EGF immunostaining was carried out using the peroxidase conjugate. Original magnification × 400. **a **– normal mice, **b **– ulcerated untreated mice, **c **– ulcerated PK-treated mice, **d **– ulcerated Omez-treated mice.

Administration of the test drugs led to an increased expression of EGF in mucosa of the ulcer margin. EGF was found to be immunolocalized in proliferative zone cells and in some parietal cells in the gastric oxyntic mucosa (Fig 5).

**Figure 6:**
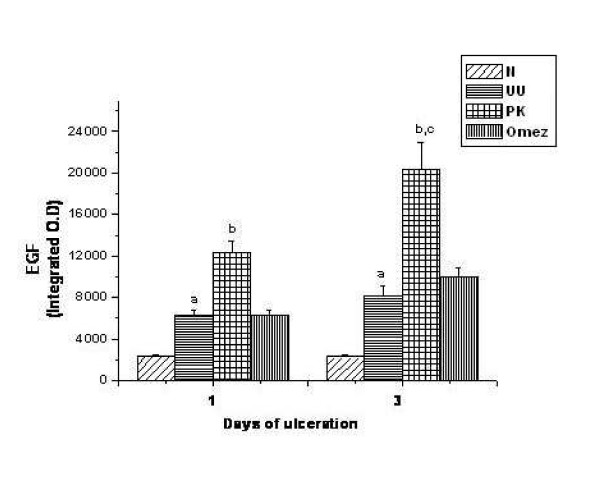
Comparative time dependent activity of PK and Omez in regulating the expression of tissue EGF in acute gastric ulcerated mice. The EGF expression was quantified using Biovis MV500 software. Data are expressed as means ± SEM for fifteen mice. ^a^*P *< 0.001 compared to the normal mice; ^b^*P *< 0.001 compared to the respective ulcerated mice, ^c^*P *< 0.01 compared to the Omez-treated mice. N – normal mice, UU – ulcerated untreated mice, PK – ulcerated PK-treated mice, Omez – ulcerated Omez-treated mice.

Compared to the normal mice, the expressions of EGF increased by ~2.7 and 3.6 folds respectively (*P *< 0.001) for the groups II and III ulcerated mice. However, treatment with PK for one and three days augmented the expression of EGF further by 98.1% and 149.0% (*P *< 0.001) compared to that in the respective ulcerated control mice. PK showed significantly better effect than Omez (*P *< 0.01), which increased the EGF expression by 11.6% compared to that of group III mice. The results are summarized in Fig. 6.

### Effect of PK and Omez on VEGF expression

**Figure 7:**
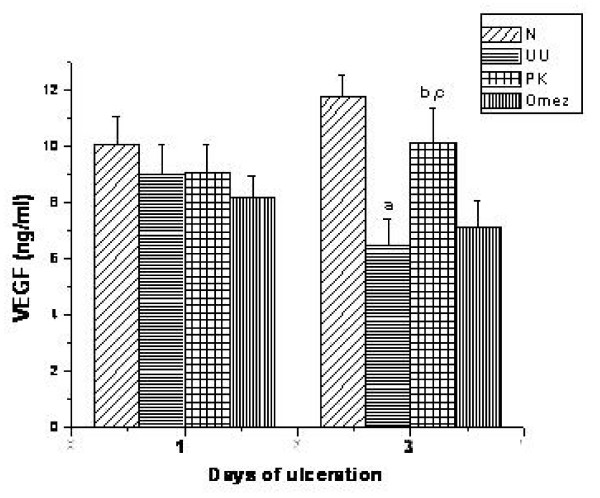
Comparative time dependent activity of PK and Omez in regulating the expression of tissue VEGF in acute gastric ulcerated mice. The EGF expression was quantified using Biovis MV500 software. Data are expressed as means ± SEM for fifteen mice. ^a^*P *< 0.01 compared to normal mice, ^b^*P *< 0.01 compared to the respective ulcerated mice, ^c^*P *< 0.01 compared to the Omez-treated mice. N – normal mice, UU – ulcerated untreated mice, PK – ulcerated PK-treated mice, Omez – ulcerated Omez-treated mice.

Compared to the normal unulcerated mice, the serum VEGF levels of the ulcerated mice was reduced by 10.3% and 44.1% (*P *< 0.01) respectively on the 1^st ^and 3^rd ^days of ulceration. Treatment with PK for 3 days increased (56.9%, *P *< 0.01) it significantly, compared to the untreated ulcerated mice (Fig. 7). The effect of PK was significantly better compared to that of Omez (*P *< 0.01).

### Effect of PK and Omez on gastric secretion

Compared to the untreated PL mice, those receiving indomethacin showed an increased volume of gastric juice (88%, *P *< 0.01) and acid output (39.8%, *P *< 0.05). Treatment with PK reduced these parameters by 18.7% (*P *< 0.05) and 19.7% (*P *< 0.05) while Omez reduced these by 43.7% (*P *< 0.01) and 32.3% (*P *< 0.05). The pH of the gastric contents that was substantially reduced by indomethacin was restored to that of the experimental control mice (Table 3).

**Table 3:** The effect of PK, Omez, and indomethacin as such and in combination with PK or Omez on the gastric secretion of pylorous ligated mice.^a ^

Mice	Gastric juice (ml) (6 h)	pH	Acid output (μEq/6 h)
Experimental control	0.85 ± 0.1	3.56	183.6 ± 6.8
Treated with PK	0.7 ± 0.1	3.41	179.2 ± 4.6
Treated with indomethacin	1.6 ± 0.2^b^	2.3	256.6 ± 10.2^a^
Treated with PK + indomethacin	1.3 ± 0.1^c^	3.46	206.2 ± 5.9^c^
Treated with Omez	0.75 ± 0.09	3.36	186.5 ± 6.8^c^
Treated with Omez + indomethacin	0.9 ± 0.12^d^	3.25	173.6 ± 6.4^c^

^a^Untreated and treated mice were subjected to pylorous ligation. The treatment groups were orally administered PK (20 mg/kg/day × 3 days), Omez (3 mg/kg/day × 3 days), indomethacin (18 mg/kg × 1 d) as such or along with PK and Omez at the designated doses. The values are mean ± SEM (n = 5). ^a^*P *< 0.05, ^b^*P *< 0.01 compared to the experimental control mice ^c^*P *< 0.05, ^d^*P *< 0.01 compared to the indomethacin-treated mice

## Discussion

Ulcer healing is a complex process involving various factors. The gastrotoxicity of the NSAIDs including indomethacin in animals can be attributed to their ability to induce the reactive oxygen metabolites. They also delay ulcer healing by reducing the prostaglandin (PG) level [2,24] and preventing the PG-mediated angiogenesis. In addition, the NSAIDs decrease gastric mucus production [25] resulting in hemorrhagic ulcer. Release of preformed mucus also plays a role in promoting epithelial recovery after acute injury by forming a mucoid cap beneath which re-epithelization occurs. The growth factors play a crucial role in ulcer healing by potentiating angiogenesis, and increasing the release of gastric mucin. Thus, drugs that arrest ulcer progression by antioxidant action, and also promote secretion of PGE_2_, gastric mucus, and growth factors would accelerate gastric ulcer healing.

Our histopathological examinations revealed that administration of indomethacin caused marked damage to the gastric mucosa within 6 h with elongated haemorrhagic lesions, confined to the glandular portion. Maximum ulcerative damage was observed on the 3^rd ^day after administration of indomethacin. The acute nature of ulceration was evident from natural recovery of the gastric tissues even without any treatment, although the process was slow.

In comparison, the mice treated with PK and Omez showed significantly faster and better healing within three days. Overall, PK and Omez C (at all doses) showed maximum efficacy on the 3^rd ^day of treatment. Interestingly, the accelerated healing by the drugs was also evident within 4 h of their administration. Under optimized treatment regime, the effect of Omez (3 mg/kg) was marginally better than that of PK (20 mg/kg). Increasing the drug dose and/or extending the treatment up to seven days led to a better healing. However, a major part of this can be attributed to natural recovery, which was substantial on the 7^th ^day of ulceration.

Tissue damage is always associated with oxidative stress leading to loss or impairment of protein synthesis and damages to lipids and the thiol-dependent antioxidant defense. Besides preventing lipid peroxidation, the sulphydryl compounds (thiols) may also protect mucus, since mucus subunits are joined by disulfide bridges that, if reduced, render mucus water-soluble [26]. In addition, they also help in recycling antioxidants like vitamin E and vitamin C. The decrease in endogenous thiol (glutathione) in ethanol induced gastric injury, and its role in mucosal protection has been demonstrated [27]. All these oxidative factors might lead to aggravated tissue damage during stomach ulceration.

Our results revealed that the stomach ulceration was associated with oxidative damages to lipids, proteins and the thiol-dependent antioxidant defense systems. This was apparent from the stimulated lipid and protein oxidation leading to increased accumulation of TBARS and protein carbonyls as well as depletion of NP-TSH. Indomethacin induced gastropathy is known to decrease the glutathione peroxidase activity and aggravate the tissue injury due to accelerated accumulation of H_2_O_2 _and lipid peroxidation. Furthermore, excessive peroxidation causes increased glutathione consumption [27]. The test samples provided a suppression of the oxidative damages to the biomacromolecules, compared to that observed in natural recovery. This might decrease the ulcer progression and promote healing of gastric lesions induced by acute intake of indomethacin.

Mucosal damage can be easily produced by the generation of reactive oxygen species. An increase in mucus production usually assist the healing process by protecting the ulcer crater against the endogenous aggressors like, stomach secretions and oxidants as well as exogenous damaging agents, such as NSAIDs. Our results revealed that stomach ulceration resulted in decreased mucin secretion. This might reduce the ability of the mucosal membrane to protect the mucosa from physical damage and back diffusion of hydrogen ions. The ulcer healing by PK and Omez was associated with an increase in the mucus layer in the gastric mucosa.

The NSAIDs are known to inhibit cyclooxygenase isoforms (COX-1 and COX-2) and decrease the PGE_2 _levels at the gastric mucosa causing ulceration [28]. Gastric injury only develops when both COX-1 and COX-2 are inhibited. Since growth-promoting actions and angiogenesis play a pivotal role in wound healing, selective cyclooxygenase-2 inhibitors may suppress these activities in gastric ulcer tissues and delay the healing process. There has been accumulating evidence that prostaglandin might contribute to ulcer healing [29].

Our immunohistochemical studies revealed reduced expression of both COX-1 and COX-2 on the day of ulcer induction as well as at peak ulceration. Treatment with PK, but not Omez for three days increased the expression of both the enzymes significantly. In tune with the above results, the concentration of mucosal PGE_2 _was also found to be reduced on the first day of ulceration itself, which was depleted further in the mice left untreated. PK augmented the mucosal PGE_2 _without showing any immediate effect. The augmentation of the mucosal PGE_2 _by PK might stimulate the EP4 receptor-mediated mucin synthesis [30], and inhibit the neutrophil-mediated free radicals generation [31].

Clinical and experimental data indicate that traditional NSAIDs delay the healing of gastroduodenal ulcers by interfering with the action of growth factors that restricts epithelial cell proliferation, maturation of the granulation tissue and impede angiogenesis [32]. The growth factor, EGF accelerates gastroduodenal ulcer healing by stimulating cell migration and proliferation in epithelial cell monolayers, tissue repair, increasing release of gastric mucin, and attenuating gastric acid secretion [33]. EGF is also reported to stimulate PG synthesis, which, in turn, augments the expression of EGF via a feedback cycle, mediated by the PKA signaling pathway [34]. The role of the endogenous EGF in ulcer healing is also unequivocally confirmed [35].

The present study revealed that gastric ulceration in mice was associated with a marked increase in the mucosal EGF level. The increase is expected since ulcer healing would require more EGF for an effective signal transduction. However, the increase was significantly lesser compared to the PK-treated mice. In comparison, Omez altered the EGF level much less. The endogenous EGF level stimulation by PK is promising for its use as an anti-ulcer agent.

Of the many growth factors, VEGF promotes endothelial proliferation and migration, and accelerate ulcer healing [35]. It promotes restoration of the connective tissue and microvessels (angiogenesis) in injured mucosa. Indomethacin inhibits ADP-induced platelet aggregation and release of the α-granule, which stores VEGF. Consequently, indomethacin treatment would reduce VEGF release. We also observed significantly reduced serum VEGF level due to ulceration that was reversed by PK, but much less by Omez.

Amongst the mechanisms involved in prostaglandin action, stimulation of mucus and cellular growth and repair as well as regulation of pH at the gastric surface are important for ulcer healing. The increased production of mucus, in turn, also enhances the binding of growth factors with their receptors [36] and contributes to healing. We found that while Omez had a strong anti-secretary activity, the effect of PK was much less. Thus, the upregulation of PGE_2 _by PK might contribute to its healing activity primarily via increasing the levels of mucin, and other growth factors, and to a lesser extent due to inhibition to gastric secretion.

Given that some drugs can show mild-to-severe side effects even after short-term intake, we also evaluated the possible toxic effect of PK on mice and found it to be non-toxic even up to a dose of 500 mg/kg. These findings suggested that PK at the dose used for ulcer-healing does not have any potential side effects in mice.

## Conclusion

Overall, the present study established that PK possesses significant healing property against indomethacin-induced stomach ulceration in mice. Its healing action could be attributed to the antioxidant activity along with the ability to modulate mucin secretion, PG synthesis, and upregulation of the growth factors. These results along with its non-toxicity suggested PK as a promising anti-ulcerogenic formulation for further evaluation. Considering the importance of angiogenesis in ulcer healing, it would be of interest to study the effect of PK on the pro and anti-angiogenic factors. Investigation in this regard is currently in progress in our laboratory and the results will be reported latter.

## Competing interests

The author(s) declare that they have no competing interests.

## Authors' contributions

DB designed the study, carried out the pharmacology experiments and statistical analysis; BM carried out the growth factor experiments; SKB participated coordination of experimental work and drafting the manuscript; SC participated in the experimental design, and preparation of the manuscript. All authors read and approved the final manuscript.

## Pre-publication history

The pre-publication history for this paper can be accessed here:


